# Laparoscopic-Assisted Resection of Jejunojejunal Intussusception Caused by a Juvenile Polyp in an Adult

**DOI:** 10.1155/2014/856765

**Published:** 2014-07-07

**Authors:** Sung Il Kang, Jeonghyun Kang, Min Ju Kim, Im-kyung Kim, Jungseob Lee, Kang Young Lee, Seung-Kook Sohn

**Affiliations:** ^1^Department of Surgery, Gangnam Severance Hospital, Yonsei University College of Medicine, 211 Enoju-ro, Gangnam-gu, Seoul 135-720, Republic of Korea; ^2^Department of Pathology, Konyang University Hospital, Daejeon, Republic of Korea

## Abstract

Most bowel intussusceptions in adults have a leading point. However, there have been few reports of jejunojejunal intussusception secondary to a solitary juvenile polyp in adult. We report herein the case of a 19-year-old female with a solitary juvenile polyp in the jejunum causing intussusception. Laparoscopic-assisted reduction and segmental resection of the jejunum were successfully done for the patient.

## 1. Introduction

Intussusception is defined by a portion of the intestine that has invaginated into another bowel loop [1]. Most intussusceptions occur in childhood and adult intussusception is rare. In contrast to intussusception in childhood, intussusception in adults usually has a leading point. Benign or malignant tumors such as lipoma, submucosal fibroma, gastrointestinal stromal tumor, Meckel's diverticulum, and adenocarcinoma can be a leading point of intussusception [2–10]. However, intussusception caused by a solitary jejunal juvenile polyp is rare. We present a case of an adult jejunojejunal intussusception caused by a solitary juvenile polyp, which was treated laparoscopically.

## 2. Case Report

**Figure 1:**
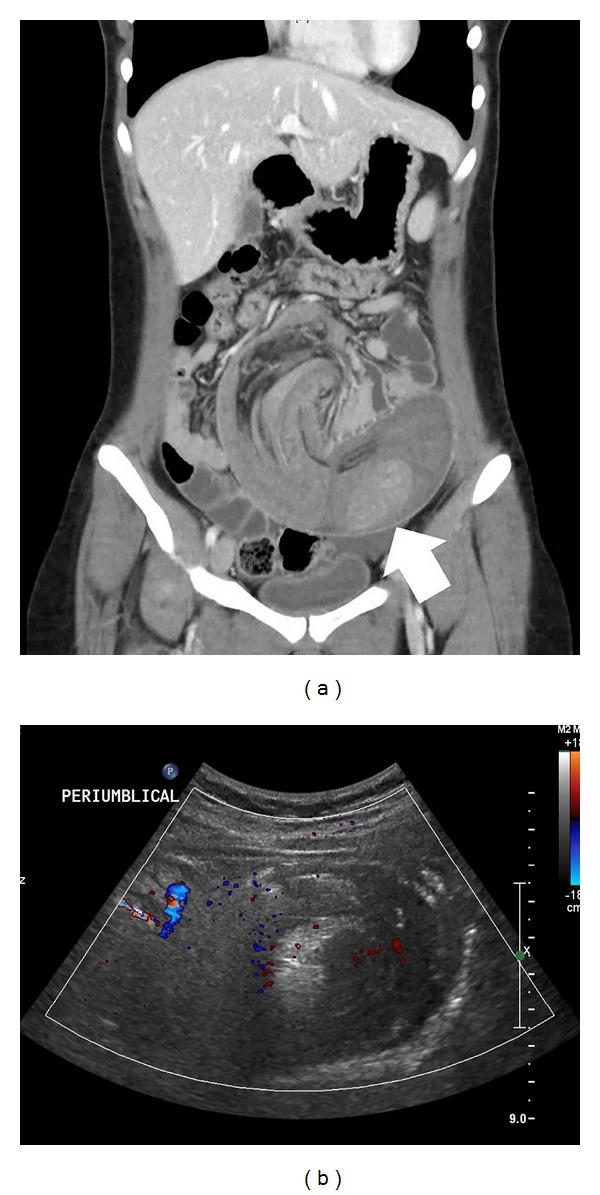
Abdominal pelvic computed tomography and ultrasonography. (a) Coronal view of the CT scan shows a long segment of jejunojejunal intussusception and polyps within the jejunum (white arrow). (b) Sonographic finding of a target sign suspected to be intussusception.

A 19-year-old female with acute developed abdominal pain of 7 hours duration was referred to our hospital from a local clinic. Abdominal contrast-enhanced computed tomography (CT) that was performed at the local clinic revealed jejunojejunal intussusception owing to a solitary polyp (Figure 1(a)). She had no specific past medical, surgical, or familial history. Her vital signs were stable. On examination, the abdomen was mildly distended, but rigidity and rebound tenderness were not clearly elicited. All laboratory findings were within the normal range. There was no evidence of definite bowel obstruction in the plain abdominal X-ray taken in our emergency department. Abdominal ultrasonography (US) was performed to evaluate the current status of intussusception. There was still long segmental small bowel intussusception with a target sign at the left periumbilical area (Figure 1(b)). We decided to perform an emergent operation.

**Figure 2:**
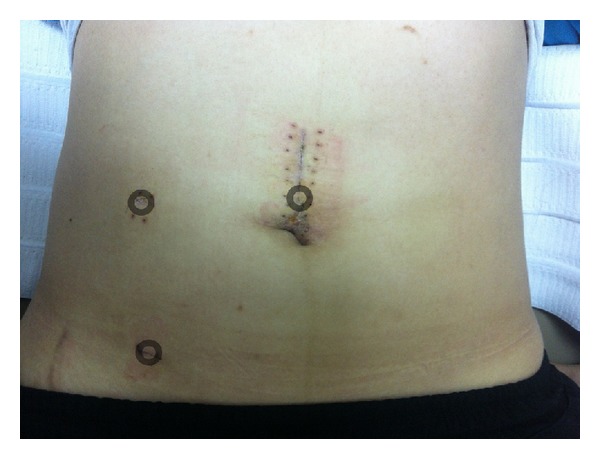
Trocar insertion sites. We used a 12 mm supraumbilical port for the camera and two 5 mm ports (O: port insertion sites).

**Figure 3:**
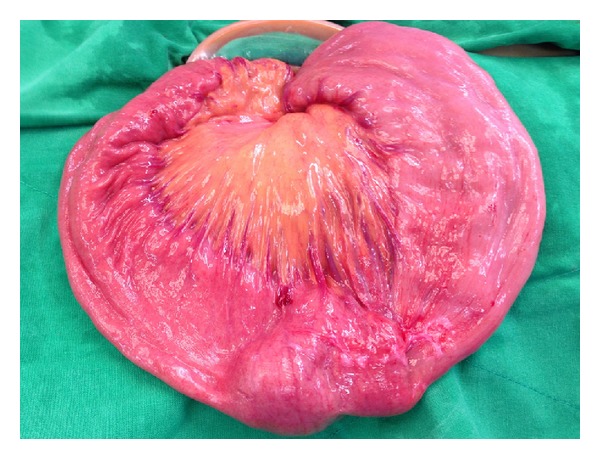
Operative findings. A polypoid mass was detected after reduction of the intussusception in the jejunum.

We used a 12 mm supraumbilical port for the camera. 5 mm ports were placed in the right mid- and lower quadrants of the abdomen (Figure 2). A jejunojejunal intussusception was found to be approximately 20 cm distal to the ligament of Treitz. The intussusceptional segment was approximately 50 cm in length. The involved bowel was dilated, but there was no evidence of bowel ischemia or perforation. Laparoscopic intracorporeal reduction with blunt graspers was performed cautiously. We palpated the remaining small bowels to the terminal ileum using laparoscopic instruments, showing no other masses or abnormalities. Afterwards, a 5 cm extension of the vertical incision was made along the supraumbilical port site, through which the small bowel was exteriorized. The large solitary luminal protruding polyp was located in the jejunum (Figure 3). Approximately 10 cm of jejunum including the polyp was resected. An end-to-end anastomosis was created by the hand-sewn method. The anastomosed jejunum was placed back into the peritoneal cavity. The extensional incision site was closed. A drain was inserted near the anastomosis site in the pelvic cavity. The total operation time was 137 minutes.

**Figure 4:**
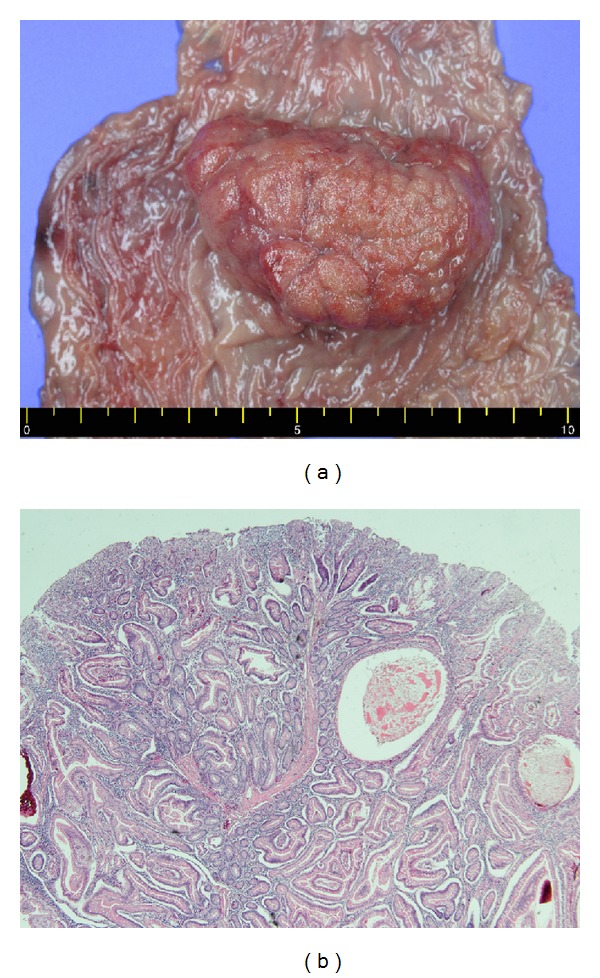
Histologic findings. (a) Gross image shows a 6 × 3.7 cm sized solitary polyp in jejunum. (b) Microscopically, the polyp showed cystic dilation and many branched tortuous glands surrounded by an inflammatory stroma, which were consistent with a juvenile polyp. Hematoxylin-eosin stain (40x).

The pathologic report indicated a solitary hamartomatous polyp, measuring 6 cm in maximum diameter (Figure 4(a)). Microscopically, the polyp showed cystic dilation and hyperplastic glands with inflammatory stroma, which were consistent with a juvenile polyp (Figure 4(b)). Since the anastomotic bowel was dilated and edematous during the operation, we advanced the patient's diet slowly. The patient was discharged on the seventh postoperative day (POD) without complications. Three weeks after the operation, the patient underwent gastroduodenoscopy, colonoscopy, and small bowel series with double-contrast barium. These examinations revealed no specific findings.

## 3. Discussion

The term “intussusception” was first used by John Hunter in 1789 to define a portion of the intestine that had invaginated into another bowel loop [1]. Most intussusceptions have a leading point such as a lipoma, submucosal fibroma, gastrointestinal stromal tumor (GIST), or Meckel's diverticulum [3, 5, 7, 10].

Jejunojejunal intussusceptions are rare. To the best of our knowledge, there have been only two reported cases of a juvenile polyp causing jejunojejunal intussusception in children in the English medical literature [11, 12]. It is reported that the peak incidence of juvenile polyps is around 4 to 5 years of age, and juvenile polyps are usually located in large intestine, especially at the rectosigmoid area [11–13]. For these reasons, a juvenile polyp causing jejunojejunal intussusception in adults is extremely rare.

Surgical resection of the involved bowel is regarded as the treatment of choice in adult intussusception, because most cases involve a leading point containing a potential malignancy. Laparotomy and “en-bloc” resection without reduction of the involved bowel have been recommended to avoid bowel perforation and seeding of potential cancer cells to other sites [8]. However, the efficacy of the reduction process during surgery remains controversial. With adequate and successful reduction and accurate diagnosis to rule out the possibility of malignancy, surgeons could minimize the range of resected bowel.

The incidence of a benign tumor or an inflammatory condition is more common than malignancy in small bowel intussusception in compared with colonic intussusception [2, 9]. In addition, imaging modalities such as CT or US are valuable for understanding the nature of the leading point [1, 4, 8, 10]. Due to the proven advantages of laparoscopic surgeries, such as minimal incision, less pain, and faster recovery, the laparoscopic approach has been increasingly adopted for the treatment of adult intussusception in recent years [1, 4, 7]. In this case, we were able to locate the leading point of the jejunojejunal intussusception by CT and US prior to surgery. Therefore, using the laparoscopic approach, we successfully performed the intracorporeal reduction of intussusception. We believe that laparoscopic approach, as compared to open procedure, could minimize the length of skin incision and resected jejunum in this young patient.

The hospital stay in this patient was 7 days. The recovery of gastrointestinal motility for this patient was somewhat delayed. The first flatus was recorded in POD 3. The delay might be associated with the dilation of long intussusceptional segment of jejunum. In part, the relatively longer hospital stay may have been associated with specific national insurance system in Korea. Because the hospital charge is smaller than expected, patients have no financial benefit from early discharge.

In conclusion, we report a rare case of a juvenile polyp in the jejunum causing jejunojejunal intussusception in an adult, which was treated by laparoscopic approach. Imaging modalities such as CT or US can contribute to the correct diagnosis and characterization of adult intussusception. Even though malignancy is a concern in adult intussusception, the treatment guidelines remain controversial. A laparoscopic approach with adequate preoperative diagnosis can provide good clinical outcomes. Therefore, surgeons can consider a laparoscopic approach first in case of small bowel intussusception in adults.
